# Penpulimab, an Fc-Engineered IgG1 Anti-PD-1 Antibody, With Improved Efficacy and Low Incidence of Immune-Related Adverse Events

**DOI:** 10.3389/fimmu.2022.924542

**Published:** 2022-06-27

**Authors:** Zhaoliang Huang, Xinghua Pang, Tingting Zhong, Tailong Qu, Na Chen, Shun Ma, Xinrong He, Dennis Xia, Max Wang, Michelle Xia, Baiyong Li

**Affiliations:** ^1^ Research and Development Department, Akeso Biopharma Co., Ltd., Zhongshan, China; ^2^ Chemical Manufacturing and Control Department, Akeso Biopharma Co., Ltd., Zhongshan, China; ^3^ Manufacturing and Quality Department, Akeso Biopharma Co., Ltd., Zhongshan, China; ^4^ Procurement and Sourcing Department and Clinical Operation Department, Akeso Biopharma Co., Ltd., Zhongshan, China; ^5^ Akeso Biopharma Co., Ltd., Zhongshan, China

**Keywords:** IgG1 anti-PD-1 antibody, Fc engineering, penpulimab, binding kinetics, immune-related adverse events

## Abstract

**Background:**

IgG4 anbibodies are deficient in stability and may contribute to tumor-associated escape from immune surveillance. We developed an IgG1 backbone anti-programmed cell death protein-1 (PD-1) antibody, penpulimab, which is designed to remove crystallizable fragment (Fc) gamma receptor (FcγR) binding that mediates antibody-dependent cell-mediated cytotoxicity (ADCC), antibody-dependent cellular phagocytosis (ADCP) and proinflammatory cytokine release.

**Methods:**

Aggregation of different anti-PD-1 antibodies was tested by size exclusion chromatography, and melting temperature midpoint (Tm) and aggregation temperature onset (Tagg) were also determined. The affinity constants of penpulimab for PD-1 and human FcγRs were measured by surface plasmon resonance and biolayer interferometry. ADCC and ADCP were determined in cellular assays and antibody-dependent cytokine release (ADCR) from human macrophages was detected by ELISA. Binding kinetics of penpulimab to human PD-1 was determined by Biacore, and epitope/paratope mapping of PD-1/penpulimab was investigated using x-ray crystallography. Additionally, patients from six ongoing trials were included for analysis of immune-related adverse events (irAEs).

**Results:**

Penpulimab demonstrated better stability and a lower level of host-cell protein residue compared with IgG4 backbone anti-PD-1 antibodies. As expected, penpulimab exhibited no apparent binding to FcγRIa, FcγRIIa_H131, FcγRIIIa_V158 and FcγRIIIa_F158, elicited no apparent ADCC and ADCP activities, and induced no remarkable IL-6 and IL-8 release by activated macrophages *in vitro*. Penpulimab was shown in the co-crystal study to bind to human PD-1 *N*-glycosylation site at N58 and had a slower off-rate from PD-1 *versus* nivolumab or pembrolizumab. Four hundred sixty-five patients were analyzed for irAEs. Fifteen (3.2%) patients had grade 3 or above irAEs. No death from irAEs was reported.

**Conclusions:**

IgG1 backbone anti-PD1 antibody penpulimab has a good stability and reduced host cell protein residue, as well as potent binding to the antigen. Fc engineering has eliminated Fc-mediated effector functions of penpulimab including ADCC, ADCP and reduced ADCR, which may contribute to its more favorable safety profile.

**Clinical Trial Registration:**

www.ClinicalTrials.gov, identifier: AK105-101: NCT03352531, AK105-201: NCT03722147, AK105-301: NCT03866980, AK105-202:NCT03866967, AK105-203: NCT04172571, AK105-204: NCT04172506.

## Introduction

Programmed cell death-1 (PD-1), a cell surface protein commonly expressed on B and T cells as well as myeloid-derived cells, is a member of the CD28 superfamily that delivers negative signals upon interaction with its two ligands, PD-L1 or PD-L2 (1). Binding of PD-1 to its ligands transduces a signal that inhibits T-cell proliferation, cytokine production, and cytolytic function (1). Anti-PD-1 antibodies, such as pembrolizumab and nivolumab, can block PD-1/PD-L1 interactions and have demonstrated impressive benefits in trials in solid tumors (2–4). Nevertheless, these drugs are not always effective, which leads to explorations in elucidating the mechanisms.

The crystallizable fragment (Fc) of IgG antibodies can bind to Fc gamma receptors (FcγRs) and trigger effector functions like antibody-dependent cellular phagocytosis (ADCP) and antibody-dependent cell-mediated cytotoxicity (ADCC). Previous study demonstrated that Fc/FcγR interactions contribute to the resistance to PD-1 blockade, as evidenced by the depletion of activated CD8 tumor-infiltrating lymphocytes (TILs) upon application of anti-PD-1 antibody with high affinity for FcγRs (5). Importantly, blockade of FcγRs before PD-1 antibody administration prolongs PD-1 antibody binding to TILs and enhances immunotherapy-induced tumor regression in mice (6). Therefore, anti-PD-1 antibodies act predominantly *via* receptor blockade, and are expected to not require Fc effector functions. However, currently marketed anti-PD-1 antibodies are typically of IgG4 isotype, which have strong FcγRI (CD64) binding and ADCP activity. PD-1 antibodies of IgG4 isotype also have residual FcγRIII (CD16) binding, and thus weak ADCC activity. Recruitment and activation of immune cells *via* these Fc receptors will subsequently lead to antibody-dependent cytokine release (ADCR) (7, 8). The secretion of proinflammatory cytokines could compromise the anti-tumor efficacy of anti-PD-1 antibody therapy and contribute to immune-related adverse events (irAEs) (9–12).

IgG1 backbone antibody is of better structure stability (13). Studies have reported that due to CH2/CH3 instability of Fc domain, IgG4 antibody is prone to aggregating with either IgG4 or IgG1 *via* Fc-Fc interaction (13, 14); moreover, anti-PD1 antibody might facilitate immune evasion of tumor cells *in vivo* by attenuating tumor-specific antibody mediated tumor killing (15–17). Furthermore, IgG4 backbone antibody tended to interact with host-cell protein (HCP). Residual HCPs, such as lipases of the host cells, have been reported to trigger immune response in humans (18).

We have developed a human IgG1 PD-1 antibody, penpulimab, also known as AK105, with Fc mutations to eliminate FcγR binding, consequently eliminating ADCC, ADCP and ADCR. In preclinical studies, we confirmed lack of effector function and proinflammatory cytokine induction by penpulimab. We also delineated antigen binding kinetics of penpulimab. In addition, we carried out a pooled analysis of irAEs of penpulimab in advanced tumor patients from six clinical trials of penpulimab to gain an initial look into the safety consequence of the differentiation antibody design.

## Materials and Methods

### HCP Residue Test

CHO HCP ELISA kit (CYGNUS, cat lot: F550-1) was used to evaluate HCP according to the kit instruction manual. The test samples were each serially diluted 4 times, and the dilution factor for each sample is shown in **Supplementary Table 1**.

Briefly, HCP quality control sample (Akeso) was diluted by Sample Diluent Buffer (CYGNUS, cat lot: I028) to 1:5000, 1:15000, and 1:45000 and served as a positive control. Calibration standards (50 μL), test sample (50 μL), assay control and anti-CHO: HRP conjugate (100 μL) were added to each well of anti-CHO coated microtiter strips (CYGNUS, cat lot: F550-1). CHO HCP standards (0 ng/mL) served as the blank. The plate was read at 450 nm and 650 nm. CHO cell protein results were calculated in ng/mL and were reported as ppm. Results >1 ppm were reported as round numbers; one significant digit was kept in the report for results <1 ppm; the results were reported as undetectable if they were below the limit of quantification (LOQ, 1 ng/mL) and were converted by using the formula: HCP = (1 (ng/mL) * dilution factor)/sample concentration (mg/mL).

### Size Exclusion Chromatography (SEC)

The pharmaceutical grade IgG antibodies used in this study are listed in **Supplementary Table 1**. The amounts of aggregates (high molecular weight species, HMWS) were analyzed by SEC using G3000SWXL column (Tosoh Corp, 7.8 mm i.d.*30 cm) (19). The mobile phase consisted of 25 mM sodium phosphate (pH 6.5) and 300 mM sodium chloride. The experimental conditions were as follows: flow rate: 0.8 mL/min; column temperature: ambient; sample temperature: ambient; run time: 20 minutes; detection wavelength: 280 nm; injection protein: 100 μg. The amounts of HMWS present in antibody samples which had been diluted into 2 mg/mL in 100 mM PBS (pH7.0) and incubated at 37°C for 3 days and 15 days in EP tubes were determined and compared to those of freshly prepared antibody samples (Day 0).

### Melting Temperature Midpoint (Tm) and Aggregation Temperature Onset (Tagg) Characterization

Tm and Tagg data of the antibodies listed in **Supplementary Table 1** were acquired using the UNit running the Client software V5.03 (20–22). A linear thermal ramp from 25 to 95°C at a rate of 1°C/min was performed. The samples were illuminated with UV Filter 1 and Blue Filter 3 with an incubation time of 180 seconds. Data analysis was achieved using the UNit Analysis software V5.03. Changes in intrinsic fluorescence (excited using the 266 nm laser) with temperature were monitored *via* analysis of the barycentric mean (BCM) between 300-430 nm. Changes in static light scattering were probed by measuring the intensities of the peaks at 266 and 473 nm, where appropriate. Tm values were obtained from the maximum gradient of the BCM *versus* temperature traces as identified by the differential of this data.

### Fortebio

The affinity constants of penpulimab, AK105 (hG1WT), a version of penpulimab with wildtype IgG1 backbone, or AK105 (hG4), an IgG4 backbone version of penpulimab to human C1q, FcγRIa, FcγRIIa (FcγRIIa_H131 and FcγRIIa_R131), and FcγRIIIa (FcγRIIIa_V158 and FcγRIIIa_F158) were measured by biolayer interferometry using ForteBio’s Octet optical biosensor (Fortebio, OctetQKe). For C1q, the antibodies (50 μg/mL) were immobilized onto FAB2G sensor, and serially diluted C1q (20 nM to 1.25 nM) flowed through the chip (60 seconds for both the binding phase and the dissociation phase) (23). For FcγRIa, the protein was immobilized on the HIS1K sensor, and serially diluted antibody (50 nM to 3.12 nM) flowed through the chip (120 seconds for both the binding phase and the dissociation phase). For FcγRIIa_R131, or FcγRIIa_H131, the proteins were immobilized on the NTA sensor, and the serially diluted antibody (200 nM to 12.5 nM) flowed through the chip (60 seconds for both the binding phase and the dissociation phase). For FcγRIIIa, FcγRIIIa_F158 or FcγRIIIa_V158 (5 μg/mL) was immobilized onto the HIS1K sensor, and the serially diluted antibody (500 nM to 31.25 nM) flowed through the chip (60 seconds for both the binding phase and the dissociation phase).

Data was acquired using Fortebio Data Acquisition 7.0 and analyzed using Fortebio Data Analysis 7.0.

### Biacore

Binding kinetics of penpulimab, nivolumab and pembrolizumab to human PD-1 extracellular domain fused to human IgG Fc domain (PD-1-hFc) were determined. PD-1-hFc was immobilized on a CM5 sensor chip. Serial dilutions of penpulimab, nivolumab or pembrolizumab ranging from 50 nM to 1.56 nM flowed through the sensor (180 seconds for the binding phase and 420 seconds for the dissociation phase). Data was acquired using Biacore Control 2.0 and analyzed using BiacoreT200 Evaluation 2.0.

### ADCC Assays

ADCC activities were determined by measuring lactase dehydrogenase (LDH) release from cells. Peripheral blood mononuclear cells (PBMCs) from a healthy volunteer were isolated using Ficoll-Paque™ Plus as instructed by the manufacturer (GE Healthcare) and cultured overnight in RPMI 1640 Complete Medium containing 5% CO_2_ at 37°C. Logarithmically growing 293T-PD1 cells were seeded into 96-well plates at 3*10^4^ cells per well and incubated with 10 μg/mL penpulimab, nivolumab or isotype control for 1 hour at room temperature. Thereafter, 4.5*10^5^ PBMCs per well were added and incubated at 37°C for 4 hours. Then, the cells were centrifuged at 250 g for 5 minutes and the supernatant was transferred to 96-well plates. Commercially available LDH assay kit (Roche) was used. Optical density (OD) was measured at 490 and 610 nm using a microplate reader (MD, PLUS384) and ADCC activity was reported as percentage of LDH release and calculated as follows: ADCC% = ((OD of the experimental group - OD of the negative control group)/(OD of the positive control group - OD of the negative control group))* 100%.

### CDC Assays

CDC activities were determined by measuring LDH release from cells. The human serum purchased from Quidel was used as the source of complements. Logarithmically growing CHO-K1-PD1-CTLA4 cells were seeded into 96-well plates at 3*10^4^ cells per well and pre-incubated with 50 μL serially diluted penpulimab, AK105 (hG1WT) or nivolumab for 10 minutes at room temperature. Thereafter, normal human serum at the final concentration of 2% were added and incubated at 37°C for 4 hours. Then, the cells were centrifuged at 250 g for 5 minutes and the supernatant was transferred to 96-well plates. Commercially available LDH assay kit (Roche) was used. OD was measured at 490 and 650 nm using a microplate reader (MD, PLUS384) and CDC activity was reported as percentage of LDH release and calculated as follows: CDC% = ((OD of the experimental group - OD of the negative control group)/(OD of the positive control group - OD of the negative control group)) * 100%.

### ADCP Assays

Bone marrow cells were obtained from the femurs of C57BL/6 mice (Guangdong Medical Laboratory Animal Center, Guangzhou, China), and induced into mature macrophages using recombinant mouse M-CSF (Peprotech). CHO-K1-PD1 cells were stained with carboxyfluorescein succinimidyl ester (CFSE) (Biolegend) and CFSE^+^ CHO-K1-PD1 cells were used as target cells. Totally 100 μL each of macrophages (5*10^2^ cells/μL), CFSE^+^ CHO-K1-PD1 cells (1.5*10^6^ cells/mL), and penpulimab (at the final concentration of 10, 1, and 0.1 μg/mL) or isotype control antibody (at the final concentration of 10 μg/mL) were aliquoted into a 1.5 mL centrifuge tube, respectively. Then, 100 μL APC-labeled goat anti-mouse CD11b antibody (Biolegend) (dilutions 1:400) was added to each sample, and incubated on ice for 40 minutes. After wash with PBS containing 1% bovine serum albumin (BSA) and centrifugation, the cells were resuspended in 200 μL PBS containing 1% BSA and transferred to BD flow sample tube. The ratio of APC^+^CFSE^+^ cells to APC^+^ cells was used as the phagocytic rate to evaluate antibody-mediated ADCP activity. ADCP% was calculated as follows: ADCP% = ((number of APC^+^CFSE^+^ cells)/(number of APC^+^ cells)) * 100%.

### ADCR From Human Monocyte-Derived Macrophages

PBMCs from a healthy volunteer were isolated by using Ficoll-Paque™ Plus as instructed by the manufacturer (GE Healthcare) and cultured overnight in RPMI 1640 medium containing 10% FBS at 37°C. Human peripheral monocyte-derived macrophages (HPMMs), derived from PBMCs, were seeded at 1×10^4^ cells per well in 96 well plates in the presence of IFN-γ (50 ng/mL). CHO-K1-PD1 cells were then seeded at 3×10^4^ cells per well. HPMMs were stimulated with penpulimab, nivolumab (BMS), pembrolizumab (MSD) or human isotype control antibody hIgG1 and hIgG4 (Akeso) at the final concentration of 0.05, 0.5 or 5 μg/mL, or 100 ng/mL LPS. After 24-hours incubation, the cells were collected and centrifuged. The supernatant was collected for determination of the concentrations of IL-6 and IL-8 in HPMMs using commercially available ELISA kits (Dakewe, 1110602/1110802) as instructed by the manufacturer. OD was read by a Molecular Devices Plus384 microplate reader.

### Receptor Occupancy

PD-1 receptor occupancy (RO) on circulating T cells was measured as an indication of target engagement. Blood samples from phase 1 subjects were used to determine PD-1 receptor occupany using a flow cytometry-based method. Patients were given penpulimab every 2 weeks (Q2W) in 4-week cycles. Details on collection of blood samples, processing, storage, and shipping details are provided in the Laboratory Manual. The time points for blood sampling for PD-1 receptor occupancy are described in **Supplementary Table 2**.

### Penpulimab to Promote PBMCs Activation

Raji-PD-L1 cells are lentivirally transfected Raji cells expressing human PD-L1. For determination of IFN-γ and IL-2 production, Raji-PD-L1 cells were seeded at 1*10^5^ cells per well in 96 well plates and incubated with 3, 30, 300, 600, 900 or 1200 nM penpulimab, nivolumab, pembrolizumab or human isotype control antibody. PBMCs from a normal subject were then added to each well at 1*10^5^ cells and the cells were collected and centrifuged after co-culture with Raji-PD-L1 cells for 3 days. The supernatant was collected for determination of IL-2 and IFN-γ concentrations with commercially available ELISA kits (Dakewe, DKW12-1020-096/DKW12-1000-096). OD was read by a microplate reader (Molecular Devices, PLUS384).

### Statistical Analysis

For *in vitro* studies, statistical analyses were performed using one-way *ANOVA* with Prism version 7 software (GraphPad Software, Inc.,La Jolla, CA). Data were expressed as number and percentage (%) or mean± standard error of mean (SEM), and *P*<0.05 was considered statistically different. Safety assessments were analyzed mainly using descriptive statistics.

## Results

### IgG1 Backbone Endues Penpulimab With Potent Stability and Good Quality Features Compared to PD-1 Antibodies With IgG4 Backbone

**Figure 1:**
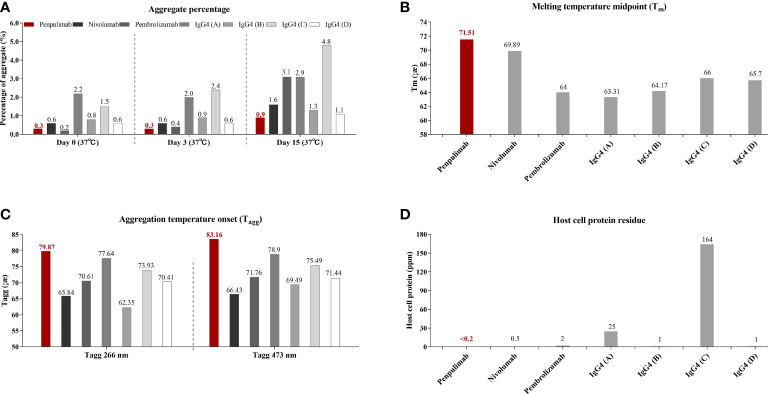
**(A)** Aggregates tested by size exclusion chromatography (SEC), **(B)** melting (Tm) and **(C)** aggregation temperature (Tagg) characterization using dynamic light scattering (DLS) and static light scattering (SLS), respectively, and **(D)** Host-cell protein (HCP) residue assay in penpulimab and other IgG4 backbone anti-PD1 antibodies.

IgG1 backbone antibody has a more stable structure from the manufacturing and long-term storage standpoint while IgG4 antibody is known for CH2/CH3 instability-mediated aggregation, which correlates to immune evasion in the tumor microenvironment in some studies (17, 24, 25). Consistent with previous studies, in aggregate assays analyzed by SEC, fewer aggregates of penpulimab were found in the dilution stability test than IgG4 backbone anti-PD1 antibody (**Figure 1A**). In addition, compared with other IgG4 backbone anti-PD1 antibody, greater conformational stability and colloidal stability of penpulimab characterized by higher Tm (**Figure 1B** **)** and Tagg (**Figure 1C**), respectively, were identified. The original images exported from the UNit running the Client software V5.03 are shown in **Supplementary Figure 1**. These data confirmed the potent stability of penpulimab as an IgG1 backbone antibody.

IgG4 backbone antibody was found prone to interacting with HCP during the manufacturing process, which causes safety concerns and accelerates degradation of formulation (18, 26). We tested HCP of penpulimab and other IgG4 backbone anti-PD1 antibodies by using commercial HCP kit. As shown in **Figure 1D**, a lower level was quantified of HCP residues of penpulimab compared with IgG4 backbone anti-PD1 antibodies.

### Fc Engineering Eliminates FcγRs and C1q Binding to Penpulimab on an IgG1 Backbone

To eliminate Fc effector functions, we introduced Fc mutations into penpulimab to eliminate FcγRs and C1q binding. As shown in **Table 1**, penpulimab did not bind to FcγRIa, FcγRIIa_H131, FcγRIIIa_F158, FcγRIIIa_V158 or C1q, and showed weak binding to FcγRIIa_R131. Meanwhile, AK105 (hG4), the IgG4 backbone penpulimab, retained binding to FcγRIa, FcγRIIa_H131, FcγRIIa_R131, and FcγRIIIa_V158.

**Table 1:** Affinity of penpulimab for FcγRIa, FcγRIIa_R131, FcγRIIa_H131, FcγRIIIa_V158, FcγRIIIa_F158 and C1q.

Sample		K_D_ (M)	kon(1/Ms)	kdis(1/s)	R_max_(nm)
**FcγRIa**	AK105 (hG4)	8.25E-09	5.79E+05	4.78E-03	0.50-0.54
	AK105 (hG1WT)	3.35E-09	6.36E+05	2.13E-03	0.60-0.67
	Penpulimab	ND	ND	ND	ND
**FcγRIIa_R131**	AK105 (hG4)	3.13E-08	3.50E+05	1.10E-02	0.21-0.46
	AK105 (hG1WT)	3.46E-08	6.60E+05	2.28E-02	0.39-0.83
	Penpulimab	2.32E-07	4.04E+05	9.38E-02	0.19-0.35
**FcγRIIa_H131**	AK105 (hG4)	5.07E-08	2.57E+05	1.30E-02	0.18-0.35
	AK105 (hG1WT)	5.74E-08	4.65E+05	2.67E-02	0.82-1.12
	Penpulimab	ND	ND	ND	ND
**FcγRIIIa_V158**	AK105 (hG4)	2.35E-05	3.56E+03	8.37E-02	2.68-8.68
	AK105 (hG1WT)	1.97E-07	1.67E+05	3.29E-02	1.50-1.66
	Penpulimab	ND	ND	ND	ND
**FcγRIIIa_F158**	AK105 (hG4)	ND	ND	ND	ND
	AK105 (hG1WT)	2.38E-07	1.68E+05	4.00E-02	0.28-0.57
	Penpulimab	ND	ND	ND	ND
**C1q**	AK105 (hG4)	ND	ND	ND	ND
	AK105 (hG1WT)	1.84E-09	5.64E+06	1.04E-02	0.26-0.39
	Penpulimab	ND	ND	ND	ND

ND, not detected. AK105 (hG1WT), a version of penpulimab with wildtype IgG1 backbone. AK105 (hG4), a IgG4 backbone version of penpulimab.

### Penpulimab Elicits No FcγRs and Complement-Mediated CDC, ADCC, ADCP or ADCR

**Figure 2:**
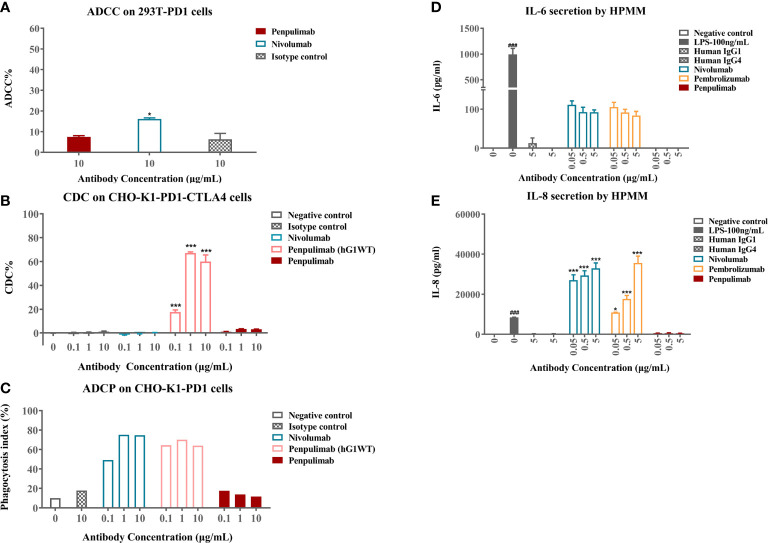
**(A)** Antibody-dependent cell-mediated cytotoxicity (ADCC) activities of penpulimab and nivolumab were determined by measuring lactase dehydrogenase (LDH) release from 293T-PD1 cells. **(B)** Complement-dependent cytotoxicity (CDC) activities of penpulimab, penpulimab (hG1WT), a version of penpulimab with wildtype IgG1 backbone, and nivolumab were determined by measuring LDH release from CHO-K1-PD1-CTLA4 cells. **(C)** Antibody-dependent cellular phagocytosis (ADCP) activities of penpulimab (hG1WT), penpulimab, and nivolumab were studied by examining phagocytosis of CHO-K1-PD1 cells by murine bone marrow derived macrophages (HPMMs). Effects of Fc engineering of penpulimab on the release of inflammatory cytokines. **(D)** IL-6 and **(E)** IL-8 by HPMMs in the presence of IFN-γ. Data are expressed as mean or mean ± SEM and analyzed using one-way *ANOVA*. *P<0.05 and ***P<0.001 *vs.* isotype control; ^###^ *P*<0.001 *vs.* negative control.

Cellular assays were conducted to confirm functional consequences of Fc engineering. In ADCC assays, nivolumab caused low levels of ADCC activity to PD-1 expressing 293T-PD1 cells as measured by LDH release from lysed cells while penpulimab exhibited no such activity (**Figure 2A**). As expected, neither showed CDC activities against PD-1-expressing CHO-K1-PD1-CTLA4 cells (**Figure 2B**). ADCP assays revealed that penpulimab caused no apparent phagocytosis to PD-1 positive CHO-K1-PD1 cells (**Figure 2C**) while nivolumab demonstrated very strong ADCP activity.

We further examined the effects of Fc engineering of penpulimab on inflammatory cytokine release by HPMMs co-cultured with PD-1-expressing cells. Penpulimab did not elicit the secretion of IL-6 or IL-8 while nivolumab and pembrolizumab triggered a strong release of these cytokines (**Figures 2D, E**). These findings together indicated that Fc engineering effectively eliminated FcγR-mediated effector function of penpulimab and remarkably abated inflammatory cytokine release from HPMMs, hinting a benign safety profile in terms of irAE occurrences.

### N-Glycan of N58 on PD-1 Is Crucial for Antigen Binding of Penpulimab

Structure of PD-1 with penpulimab using x-ray crystallography at a resolution of 2.5 Å was determined (**Supplementary Figure 2**). Complex structures of PD-1 with nivolumab or pembrolizumab Fab have been reported, and, surprisingly, we discovered that AK105 could bind to PD-1 with different orientations from pembrolizumab and nivolumab, and more importantly, penpulimab showed numerous contacts with N58 glycosylation on the BC loop of PD-1 (**Supplementary Figure 3**), which are not found in pembrolizumab and nivolumab (1, 27).

### Penpulimab Exhibits Slower Binding Off-Rate for Human PD-1

**Figure 3:**
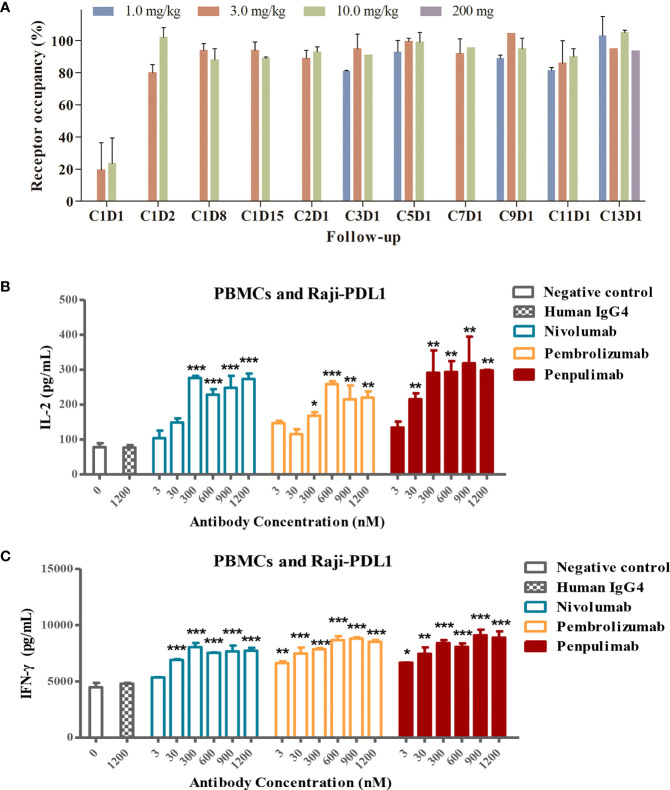
**(A)** PD-L1 target occupancy following intravenous administration of 1.0, 3.0 or 10.0 mg/kg penpulimab once every two weeks (Q2W) in the dose escalation study or 200 mg penpulimab Q2W in the expansion study, with 28 days per cycle. Post-infusion blood samples were collected at day 1, 2, 8, and 15 of cycle 1, and day 1 of cycle 2, 3, 5, 7, 9, 11, and 13, respectively, as indicated on the *x*-axis. C: cycle; D: day. Penpulimab potentiates T cell activation *via* PD1/PDL1 blockade. Raji-PD-L1 cells overexpressing PD-L1 were co-cultured with peripheral blood mononuclear cells (PBMCs) from a normal subject, and the production of IL-2 **(B)** and IFN-γ **(C)** was examined by ELISA. Data are shown as mean ± SEM for *n* = 2, and analyzed using one-way *ANOVA*. *P<0.05, **P<0.01 and ***P<0.001 *vs.* isotype control.

Head-to-head comparison was made of kinetic parameters of penpulimab, nivolumab and pembrolizumab binding to PD-1-hFc. In the assays in which human PD-1 was immobilized onto the sensor of Biacore, a slower off-rate of penpulimab *versus* nivolumab or pembrolizumab was observed (**Table 2**). Furthermore, in line with its slower off-rate, the PD-1 receptor occupancy rate of penpulimab reached 80% to 100% two days after penpulimab infusion (1.0 to 10.0 mg/kg, Q2W). After multiple infusions, the PD-1 receptor occupancy rate ranged between 80% and 100% (**Figure 3A**), suggesting nearly full target engagement at the tested doses. These findings together showed that anti-PD-1 antibody penpulimab possessed great potency and high affinity for human PD-1, which could be partially attributed to unique antigen binding epitopes of penpulimab.

**Table 2:** Affinity of penpulimab, nivolumab and pembrolizumab for PD1-hFc.

Antigen	Method	Antibodies	K_D_ (M)	kon (1/Ms)	kdis (1/s)	R_max_ (RU)
**PD1-hFc**	Biacore	Penpulimab	5.88E-10	1.62E+05	9.51E-05	32.52-46.80
		Nivolumab	5.40E-10	4.50E+05	2.43E-04	33.97-48.01
		Pembrolizumab	7.17E-10	3.91E+05	2.80E-04	44.84-63.91

### Penpulimab Potentiates T Cell Activation *via* PD-1/PD-L1 Blockade

PD-1 blockade by anti-PD-1 antibodies promotes T-cell responses and elevates cytokine secretion. We examined the effects of PD-1/PD-L1 blockade by penpulimab on IFN-γ and IL-2 production by T cells. Raji-PD-L1 cells overexpressing PD-L1 were cultured with PBMCs in the presence of penpulimab, nivolumab, pembrolizumab or isotype control antibodies. Penpulimab significantly increased IL-2 and IFN-γ production by PBMCs (**Figures 3B, C**).

### IrAEs

To examine the effects on clinical safety of the Fc engineering design on an IgG1 backbone for penpulimab, we analyzed irAEs in patients who received penpulimab during clinical trials. The population consisted of totally 465 patients, with 77 patients who received 200 mg penpulimab Q3W in the combination treatment group, 372 patients (penpulimab 200 mg Q2W) including 94 refractory/relapsed (R/R) classic Hodgkin lymphoma (cHL) patients and 83 Australian patients, as well as 16 solid tumor patients who received 1, 3, and 10 mg/kg penpulimab Q2W (**Supplementary Figure 4**). IrAEs were observed in 126 (27.1%) patients (**Table 3**). CTCAE grade 3 and above irAEs occurred in 4.3% R/R cHL patients, 2.4% patients receiving 200 mg penpulimab Q2W and 3.2% all patients receiving penpulimab. Among 465 patients, CTCAE grade 3 irAEs included immune-related hepatitis (0.6%), immune-related cutaneous adverse reaction (1.3%), immune-related pneumonitis (0.6%), immune-related hypophysitis (0.4%) and immune-related nephritis (0.2%). No irAEs that had been reported for other PD-1/PD-L1 antibodies occurred in our patients, including hematoxicities, cardiotoxicities, ocular toxicities, toxicities of the muscular skeletal system and pancreatic toxicities. No CTCAE grade 4 or 5 irAEs were reported. Twelve (2.6%) patients had serious irAEs. No death due to irAEs was reported and 9 (1.9%) patients discontinued penpulimab treatment. Twenty-seven (5.8%) patients experienced treatment interruptions due to irAEs. Eight (8.5%) patients with R/R cHL had irAEs that caused treatment interruptions and 4.3% patients discontinued treatment because of irAEs.

**Table 3:** Immune-related adverse events (irAEs) in the study population.

Preferred terms	R/R cHL*200 mg Q2W(N=94)	200 mg Q2W			200 mg Q3W in combination with other drugs^#^(N=77)	200 mg and other doses^$^(N=465)
		Phase Ib* (Australia)(N=83)	Chinese patients including cHL patients*(N=289)	All patients(N=372)		
**All irAEs**	51 (54.3%)	12 (14.5%)	94 (32.5%)	106 (28.5%)	13 (16.9%)	126 (27.1%)
**Serious irAEs**	3 (3.2%)	0	8 2.8%)	8 (2.2%)	3 (3.9%)	12 (2.6%)
**irAEs causing death**	0	0	0	0	0	0
**irAEs causing treatment interruptions**	8 (8.5%)	1 (1.2%)	20 (6.9%)	21 (5.6%)	5 (6.5%)	27 (5.8%)
**irAEs causing treatment discontinuation**	4 (4.3%)	0	6 (2.1%)	6 (1.6%)	3 (3.9%)	9 (1.9%)
**CTCAE grade 3 or above irAEs**	4 (4.3%)	0	9 (3.1%)	9 (2.4%)	4 (5.2%)	15 (3.2%)
** Hepatotoxicities (hepatitis)**	0	0	2 (0.7)	2 (0.5)	0	3 (0.6)
ALT elevations	0	0	1 (0.3)	1 (0.3)	0	2 (0.4)
AST elevations	0	0	1 (0.3)	1 (0.3)	0	2 (0.4)
Transaminase abnormalities	0	0	1 (0.3)	1 (0.3)	0	1 (0.2)
** Cutaneous toxicities**	2 (2.1)	0	4 (1.4)	4 (1.1)	2 (2.6)	6 (1.3)
Psoriasis	1 (1.1)	0	1 (0.3)	1 (0.3)	0	1 (0.2)
Rashes	1 (1.1)	0	3 (1.0)	3 (0.8)	1 (1.3)	4 (0.9)
Generalized rashes	0	0	0	0	1 (1.3)	1 (0.2)
** Pulmonary toxicities (pneumonia)**	1 (1.1)	0	2 (0.7)	2 (0.5)	1 (1.3)	3 (0.6)
Immune mediated pneumonitis	1 (1.1)	0	2 (0.7)	2 (0.5)	0	2 (0.4)
Pulmonary inflammation	0	0	0	0	1 (1.3)	1 (0.2)
** Nephrotoxicities (nephritis and renal insufficiency)**	1 (1.1)	0	1 (0.3)	1 (0.3)	0	1 (0.2)
Other nephrotoxicities	1 (1.1)	0	1 (0.3)	1 (0.3)	0	1 (0.2)
Kidney injury^a^	1 (1.1)	0	1 (0.3)	1 (0.3)	0	1 (0.2)
** Endocrine toxicities**	0	0	0	0	1 (1.3)	2 (0.4)
** Hypophysitis**	0	0	0	0	1 (1.3)	2 (0.4)

Data are expressed as N(%).

*R/R cHL patients in the AK105-201 study, Australian patients in the AK105-101 phase 1b study, and Chinese patients from Ak105-201, AK105-202, and AK105-204 trials.

^#^Patients from AK105-203 and AK105-301 (part I).

^$^Patients from AK105-101 (Phase 1a and 1b), AK105-201, AK105-202, AK105-203, AK105-204 and AK105-301 (part I).

cHL, classic Hodgkin lymphoma; CTCAE, Common Terminology Criteria for Adverse Events; irAEs, immune related adverse events; Q2W, once every two weeks; Q3W, once every three weeks; R/R cHL, refractory/relapsed cHL.

a IgA nephropathy, refractory/relapsed cHL.

## Discussion

The S228P hinge-stabilization mutation of IgG4 has solved the half-antibody issue of the IgG4 backbone (28). However, weakened non-covalent binding of the CH2/CH3 in Fc region still exists, which may cause an Fc-Fc interaction (13, 14). It was thought that IgG4 antibody is a protective antibody of the body, which can dynamically exchange half-molecules, resulting in effectively monovalent antibodies that can bind to other IgG molecules in the body through Fc-Fc interaction, thus inhibiting the humoral immune response and maintaining immune tolerance (15). However, patients with cholangiocarcinoma, esophageal cancer, melanoma and other malignant tumors have found significant increases in endogenous IgG4. These endogenous IgG4 may bind to tumor-specific IgG1 in the tumor microenvironment through Fc-Fc interaction, blocking tumor-specific IgG1-mediated ADCC, CDC, or ADCP to tumor cells and promoting tumor cell immune escape (16). For an anti-PD1 antibody of IgG4 backbone, S228P mutation did not seem to completely prevent Fc-Fc interation and it was found to inhibit anti-tumor humoral immune response by Fc-Fc interaction with endogenous tumor-specific IgG1 or IgG4, which even promote tumor growth and result in hyperprogressive disease (17).

Moreover, in antibody manufacturing process, antibodies are expressed by host cells that are usually the CHO cell system and purified by Protein A chromatography to obtain the antibodies. Residual HCPs that are exogenous antigens for humans after purification are found to be associated with allergic reaction such as infusion reaction. It has been reported that during the antibody purification process of Protein A chromatography, PLBL2, a HCP in CHO cell, binds preferentially to IgG4 antibodies compared to IgG1 antibodies (18). Consistent with these findings, data from our team also identified the lowest level of HCP in penpulimab formulation compared with commercial IgG4 anti-PD1 antibody drugs. Therefore, we believe the selection of IgG1, as opposed to IgG4, as the backbone for oncology therapeutic antibodies, may have intrinsic advantages.

The evolution of different isotypes of antibody serves designated physiological purposes. The selection of IgG4 as the carrier backbone for PD-1 antibody was regarded as picking the most suitable from what is offered by nature. However, IgG4 *via* binding to FcγRs recruit macrophages and neutrophils to be in proximity to PD-1-expressing T lymphocytes. It was shown that engagement of activating FcγRs by PD-1 antibodies with IgG4 backbone leads to depletion of TILs *in vitro* and *in vivo*, abrogating therapeutic activity (5). Most of the PD-1 antibodies, including nivolumab and pembrolizumab, have IgG4_S228P_ heavy chain, which are likely retain the binding to FcγRI. On the one hand, ADCP effect may eliminate these presumably tumor fighting T cells, and on the other hand, secretion of proinflammatory cytokines by these cells may cause both a more immune suppressive environment and exacerbate irAEs (6, 8, 29).

PD-1 checkpoint inhibitors have been reported to be associated with a specific spectrum of toxicities and irAEs due to their immune mechanism of action. An important but, to some extent, overlooked mechanism, ADCR, which is induced by an Fc/FcγR interaction between antibody-opsonized cells and immune cells, has been found to contribute to the safety and efficacy of an immune-checkpoint therapeutic antibody. FcγRIa was identified to play a key role in mediating proinflammatory cytokine release through the activation of immune cells, especially macrophages, by FcγRIa/Fc interaction, and result in a significant increase of IL-6, IL-8, TNF-ɑ and other cytokines (6, 8). IL-8, a pro-tumorigenic and immune suppressive cytokine secreted by activated macrophage, was found to associate with attenuated efficacy of PD-1 antibody therapy. *In vitro* and *in vivo* studies demonstrate that IL-8 promotes tumor cell migration, invasion and metastases. Furthermore, by recruiting immunosuppressive cells into the tumor, IL-8 contributes to tumor immune evasion. In cancer patients, higher levels of IL-8 predicted a worse clinical outcome. In patients treated with immune checkpoint inhibitor, IL-8 contributes to resistance to immune checkpoint inhibitor. Furthermore, pre-clinical studies indicate that blockade of IL-8 improved anti-tumor immune response, and combination of anti-IL8 treatment with immune checkpoint inhibitor improve anti-tumor efficacy (30, 31).

Tislelizumab (BGB-A317) is a PD-1 antibody that also has been engineered to remove FcγR binding. *In vitro* study showed that IL-6 and IL-8 production after treatment with anti-PD-1/IgG4_S228P_ antibodies were higher than that with tislelizumab (32). Thus, removing FcγR binding provides additional rationale for developing penpulimab, which is designed to contain triple mutations in Fc region to eliminate effector functions. Fc engineering does not impact on half-life of penpulimab. Based on existing population PK data, the mean elimination half-life ( ± SD) of penpulimab is 23.3 ± 9.51 days, which is similar to that of pembrolizumab (33).

Glycans play an important role in cancer, such as cell signaling and communication, tumor cell dissociation and invasion, cell-matrix interactions, tumor angiogenesis, immune modulation and metastasis formation as well as immune surveillance (34). There are four reported glycosylation sites within the extracellular immunoglobulin variable (IgV) domain of PD-1, namely, N49, N58, N74, and N116 (35–37). N58, which is on the BC loop of PD-1 and resides closest to the binding epitopes of pembrolizumab and nivolumab, was reported to be heavily glycosylated and most of the glycans consisted of two *N*-acetylglucosamines (GlcNac) and one fucose in the core position when PD-1 was expressed in both mammalian and insect cells (36, 38). Fucosylation has been associated with cancers, and exhausted T cells in tumors carried highly core-fucosylated structures (35, 39). Overexpression of FUT8 and core fucosylation was observed in several cancers, such as lung and breast cancers (40, 41). The involvement of possible core fucoses in binding could be an advantage to facilitate interaction of antibodies with PD-1 with enhanced binding affinity and may lead to better clinical results (42). For penpulimab, glycosylation dependent binding to PD-1 brings a more potent antigen binding activity and robust pharmacological activity. PD-1 blockade enhances T-cell responses and promotes cytokine secretion. IFN-γ is a key cytokine produced by activated T cells and has been implicated in promoting anti-PD-1 antitumor activities (43, 44). This study showed that similar to nivolumab, penpulimab significantly promoted IFN-γ and IL-2 production by T cells *in vitro*. Furthermore, our preclinical studies showed that penpulimab (8 mg/kg and 10 mg/kg body weight) had potent antitumor activities in human PD-1 knock-in tumor model bearing colorectal adenocarcinoma xenografts and SCID/beige mice model subcutaneously inoculated with Raji-PD-L1 cells plus freshly isolated human peripheral blood mononuclear cells (data not shown).

In our phase 1a study, the ORR was 28.6% and the DCR was 57.1%. Remarkably, one pancreatic carcinoma patient and one bile duct cancer patient receiving 1.0 mg/kg penpulimab Q2W achieved PR, with a duration of response >96 weeks (45). In a phase II study, penpulimab attained an objective response rate (ORR) of 89.4% in R/R cHL patients who had received at least two prior lines of therapy, with 47.1% of the patients achieving complete response (CR). The median duration of follow up was 15.8 months and the 12-month progression-free survival rate was 72.1% and the 18-month overall survival (OS) reached 100% (46). Penpulimab also had an ORR of 29.7% and a disease control rate of 49.5% for nasopharyngeal carcinoma patients who had received at least two prior lines of chemotherapy. At a median follow up duration of 14.7 months, the median time to remission was 1.8 months and the median duration of response was still immature. The median PFS was 3.7 months as assessed by IRRC and the median OS was 18.6 months (47). These findings suggest that penpulimab is a promising immunotherapeutic agent and can lead to durable responses in certain types of advanced tumors.

By analyzing the irAEs of 465 patients from six clinical trials of penpulimab in advanced tumor patients, a favorable immune-related safety profile was identified. The mechanisms underlying the development of irAEs are rather complicated. By reducing the release of IL-6, IL-8, and other cytokines, penpulimab reduced the severity of irAEs. The rate of grade 3 and above irAEs, especially immune-related inflammatory reaction, is lower with penpulimab. These irAEs are of particular concern for immune checkpoint inhibitors (ICIs) in clinical practice as they are difficult to manage, lead to treatment interruptions or discontinuations, and may eventually result in death. The rate of grade 3 and above irAEs was 3.2% with penpulimab and the rate of irAEs leading to treatment discontinuations was 1.9%. No grade 4-5 irAEs were reported. A systemic review showed that the rate of grade 3/4 irAEs with CTLA-4 and PD-1 inhibitors was 31% and 10%, respectively, and the rate of irAEs leading to treatment discontinuation was 3%-25% and 3-12%, respectively. The rate of irAEs leading to death was 0.3% with nivolumab and 0.1% with pembrolizumab; deaths occurred mostly due to pneumonitis (48). The rate of grade 3 and above immune-related pneumonitis, enteritis, hepatitis and nephritis was 0.9%, 1.7%, 1.5% and 0.5% with nivolumab, respectively, and 1.3%, 1.1%, 0.4% and 0.1% with pembrolizumab, respectively (49, 50). The rate of grade 3 and above immune-related pneumonitis and hepatitis was 0.6% with penpulimab. No grade 3 and above immune-related enteritis, nephritis, myocarditis, and pancreatitis were observed with penpulimab. Though we cannot make a head-to-head comparison and the data was based on different patient populations, penpulimab is indeed associated with a lower rate of grade 3 and above irAEs, which may help with long-term and safe use of the drug in patients. The incidence of irAEs in R/R cHL patients was higher than that of patients treated with 200 mg penpulimab Q2W and other groups of patients, which may be related to higher rates of thyroid toxicities in R/R cHL patients. All thyroid toxicities were CTCAE grade 1-2. When thyroid toxicities were excluded, the incidence of irAEs was similar among the groups. R/R cHL patients received thyroid function test every two weeks, which may explain their higher incidence of irAEs than that of other groups of patients.

The study has several limitations. Though our preclinical studies attempted to provide an explanation for irAEs associated with penpulimab in terms of abolished ADCP, ADCR and ADCC, we did not investigate immune mechanisms underlying the action and irAEs of penpulimab. The safety data of the current study was pooled from several clinical trials that lack a controlled arm.

Currently marketed anti-PD-1 antibodies are typically of IgG4 isotype, which has residual ADCC and intact ADCP, and ADCR effects. Studies have shown that these effector functions compromise anti-tumor activity of anti-PD-1 antibody and may contribute to irAEs in patients treated with anti-PD-1 antibody. Moreover, due to instability of antibody structure, an Fc-Fc interaction of IgG4 antibody attenuates its anti-tumor activity, and even promotes disease progress. Penpulimab is an IgG1 anti-PD-1 antibody with stable structure, and elimination of binding to FcγRIa, FcγRIIa and FcγRIIIa by Fc-engineering, thus avoiding ADCC, ADCP and ADCR effects. Penpulimab also features a slower PD-1 binding off-rate through contacts with N58 glycosylation, which likely contributes to more complete receptor occupancy in patients. The cellular and clinical safety consequences of Fc-engineering is examined and revealed a more favorable profile. The selection of IgG1 isotype and optimal binding feature for checkpoint blocking therapeutic antibodies could have significant implications for clinical efficacy and safety and worth careful consideration. The results of this comprehensive study show that penpulimab has a favorable immune-related safety profile.

## Data Availability Statement

The raw data supporting the conclusions of this article will be made available by the authors, without undue reservation.

## Ethics Statement

The studies involving human participants were reviewed and approved by Icon Cancer Centre, St Vincent’s Hospital Sydney, Chinese PLA General Hospital and Medical School, Fudan University Shanghai Cancer Center, The First Affiliated Hospital, Zhejiang University School of Medicine, Peking University Cancer Hospital and Institute. The patients/participants provided their written informed consent to participate in this study. The animal study was reviewed and approved by Akeso Biopharma Animal Experiment Ethics Committee.

## Author Contributions

Conceptualization, MX, BL, MW, and DX. Resources, MX, BL, MW, and DX. Data curation, MX, BL, and MW. Formal analysis, BL, MW, ZH, XP, TZ, TQ, SM, and XH. Supervision, MX, BL, MW, and DX. Validation, ZH, XP, TZ, TQ, SM, and XH. Investigation, MX, BL, MW, and DX. Visualization, BL and NC. Methodology, BL, MW, ZH, XP, TZ, TQ, SM, and XH. Writing - original draft, BL and NC. Project administration, BL, MW, ZH, XP, TZ, TQ, SM, and XH. Writing - review and editing, BL and NC. All authors contributed to the article and approved the submitted version.

## Conflict of Interest

All authors are employees of Akeso Biopharma Co., Ltd., who participated in the discovery, production and commercialization of penpulimab.

## Publisher’s Note

All claims expressed in this article are solely those of the authors and do not necessarily represent those of their affiliated organizations, or those of the publisher, the editors and the reviewers. Any product that may be evaluated in this article, or claim that may be made by its manufacturer, is not guaranteed or endorsed by the publisher.
