# Supplementary material for: Penpulimab, an Fc-Engineered IgG1 Anti-PD-1 Antibody, With Improved Efficacy and Low Incidence of Immune-Related Adverse Events
Source: Front Immunol. 2022 Jun 27;13:924542. doi: 10.3389/fimmu.2022.924542 (PMC9272907; doi:10.3389/fimmu.2022.924542)
Supplement: Supplementary file 1 [file DataSheet_1.docx]

**Supplementary Materials**

**Methods**

***Penpulimab-Fab-his/human PD-1-his complex preparation and crystallization***

Complex of penpulimab-Fab-his/PD-1-his were obtained by mixing the two at a molar ration of 1:2, and incubated on ice for 2 hours. Then mixture was purified by SuperdexTM 200 10/300 GL (GE Healthcare). Purified penpulimab-Fab-his/PD-1-his proteins were then concentrated to 10 mg/ml. Crystals of penpulimab-Fab/PD-1 complex were grown in PEGⅡsuit F2 (0.1M MES, pH 6.5, 20% PEG4000, 0.6M sodium chloride). Crystals were flash-cooled in liquid nitrogen after incubating in anti-freezing buffer (reservoir solution containing 30% (v/v) EG). Crystal was recovered and identified to be PD1-His+AK105-Fab-His complex by SDS-PAGE.

***Data collection and structure determination***

Diffraction data of crystals were collected at Shanghai Synchrotron Radiation Facility (SSRF). All data were processed with DIALS after collection^1^. Aimless was used to implement scaling models, and Molrep was used to find the phase solution in two steps in molecular replacement method. 5% of data was randomly select for R free estimation. PD-1 structure (PDB: 3rre) and antibody structure (PDB: 6foe) were used to search models^2,3^. Then REFMAC5 is used to modify the model in the reciprocal space. Use COOT to correct the protein model in real space. After multiple cycles, the model is in good agreement with the electron density map, and the crystallographic R factor and R free are 0.21 and 0.27, respectively.

***Ethical statements***

The study protocols for the following trials AK105-101 phase 1a and 1b, AK105-201, AK105-202, AK105-204, AK105-203 and AK105-301 (part I) were approved by the ethics committees of all participating institutions and adhered to the SPIRIT statement^4^. The trials are registered with ClinicalTrials.gov (AK105-101: NCT03352531; AK105-201: NCT03722147; AK105-301: NCT03866980; AK105-202：NCT03866967; AK105-203: NCT04172571; AK105-204: NCT04172506) and were conducted in accordance with the Declaration of Helsinki. All study subjects provided written informed consent.

The study protocols for animal studies were approved by Institutional Animal Care and Use Committee (IACUC) of Crown Bioscience Inc. (approval No.: AN-1702-007-3 for mouse study), which is fully accredited by the Association for Assessment and Accreditation of Laboratory Animal Care International (AAALAC). Animal study was carried out in strict accordance with institutional and state guidelines on the experimental use of animals.

***Patients and treatment***

Patients from six datasets were included in safety analysis. The AK105-101 phase 1a trial was a multicenter open-label dose escalation study that enrolled adult patients (≥18 years) with pathologically or cytologically proven advanced or metastatic solid tumors who relapsed/were refractory to standard therapies or for whom no effective standard therapy was available, or the subject refused standard therapy. The study eligibility criteria are detailed in Supplementary Methods. Dose escalation proceeded based on a 3 + 3 design and was determined by the Dose Escalation Committee according to safety and PK/pharmacodynamics (PD). Patients received 1.0, 3.0 or 10.0 mg/kg penpulimab once every two weeks (Q2W), with each treatment cycle lasting for 28 days. At least three and maximally six evaluable patients were required for each dose (1.0, 3.0 or 10.0 mg/kg once Q2W). If maximum tolerated dose (MTD) was not reached in the low and mid dose groups, six evaluable patients were included in the highest dose group.

In addition, refractory/relapsed classic Hodgkin lymphoma (cHL) patients in the AK105-201 study, Australian patients in the AK105-101 phase 1b study, and Chinese patients from Ak105-201, AK105-202 and AK105-204 trials received 200 mg penpulimab Q2W. Furthermore, patients from AK105-203 and AK105-301 (part I) trials received 200 mg penpulimab once every three weeks (Q3W). Patients in AK105-101 (Phase 1a and 1b), AK105-201, AK105-202, AK105-203, AK105-204 and AK105-301 (part I) received 200 mg penpulimab and other doses of the drug.

AK105-301 is a phase 3, randomized, double-blinded, multicenter study of AK105 combined with carboplatin and pemetrexed vs placebo combined with carboplatin and pemetrexed as first-line therapy in patients with metastatic nonsquamous non-small cell lung cancer. Other 5 trials are phase 1 or phase 2 trials without blinding. No power calculation was done in the current analysis. Furthermore, sex was not a biological variable in this study. Ninety-four patients were included in the 200 mg Q2W R/R cHL cohort. Their mean age was 34.8 ± 12.3 years (range 18, 69). They included 59.6% men and 40.4% women, and 96.8% patients were Han Chinese. Their body weight was 67.3 ± 14.4 kg (range 40.0, 113.5). The 200 mg Q2W cohort consisted of 83 patients from Australia phase Ib trial and 289 Chinese patients including cHL patients. Their mean age was 50.5 ± 15.6 years (18, 91). They included 64.0% men and 36.0% women, and 75.3% patients were Han Chinese and 18.0% were Caucasian Australians. Their body weight was 63.9 ± 13.5 kg (range 38.5, 113.5). The 200 mg Q3W combination cohort enrolled 77 patients, including 74% men and 26% women. Their mean age was 58.8 ± 10.3 (range 24, 74) and 94.8% of them were Han Chinese. Thie mean body weight was 63.5 ± 13.1 kg. The cohort of patients receiving 200 mg and other doses enrolled 465 patients, including 64.1% men and 35.9% women, with a mean age of 52.3 ± 15.5 years (range 18, 91). In addition, 75.9% of them were Han Chinese and 17.4% were Caucasian Australians. Their body weight was 64.0 ± 13.5 kg (range 38.5, 113.5).

***Assessment of immune-related adverse events***

Adverse events (AEs) were graded and recorded according to the National Cancer Institute Common Terminology Criteria for Adverse Events (NCI-CTCAE) version 4.03. Safety events included AEs and severe adverse events (SAEs). IrAEs that were consistent with immune-related causes were observed and recorded from the date of the first dose to 30 days after the last dose for non-severe irAEs and 90 days after the last dose for severe irAEs. Severe AEs (SAEs) included any untoward medical occurrence that resulted in death, was life-threatening, required hospitalization or prolongation of hospitalization, or caused significant or persistent disability or incapacity, or birth defects. AEs were coded to a preferred term using the Medical Dictionary for Regulatory Activities (MedDRA) 22.0. Safety assessments were based mainly on the occurrence, frequency, and severity of AEs. For all AEs, where necessary, patients were withdrawn from the study. Safety analysis included all patients who received at least one dose of the study drug and had at least one follow-up safety assessment. Safety assessments were analyzed mainly using descriptive statistics.

**Supplementary references**

1. Evans PR, Murshudov GN. How good are my data and what is the resolution? Acta Crystallogr D Biol Crystallogr. 2013;69 (Pt 7):1204-1214.
2. Collaborative Computational Project, Number 4. The CCP4 suite: programs for protein crystallography. Acta Crystallogr D Biol Crystallogr. 1994;50(Pt 5):760-763.
3. Read J, Pearce J, Li X, Muirhead H, Chirgwin J, Davies C. The crystal structure of human phosphoglucose isomerase at 1.6 A resolution: implications for catalytic mechanism, cytokine activity and haemolytic anaemia. J Mol Biol. 2001;309(2):447-463.
4. Chan AW, Tetzlaff JM, Altman DG, Laupacis A, Gøtzsche PC, Krle A-Jerić K, et al. SPIRIT 2013 Statement: defining standard protocol items for clinical trials. Rev Panam Salud Publica. 2015;38(6):506-514.

**Supplementary figure legends**

**Supplementary Figure 1**. **Comparison the (A)** **melting (Tm) and (B) aggregation temperature (Tagg) between penpulimab and other IgG4 backbone commercial PD-1 antibodies (°C).** In (A), changes in intrinsic fluorescence with temperature were monitored *via* analysis of the barycentric mean (BCM) between 300-430 nm Tm values were obtained from the maximum gradient of the BCM *versus* temperature traces as identified by the differential of this data.

**Supplementary Figure 2. Overall structure of PD-1 with AK-105-Fab complex.** The Fab fragment of AK105 is shown as cartoon (Heavy chain, slate; Light chain, violet), and PD-1 is shown as surface representation (tv-yellow).

**Supplementary Figure 3. Distinct blockade binding modes of AK105 from pembrolizumab and nivolumab.** (A) Superposition of PD-1-nivolumab-Fab, PD-1-pembrolizumab-Fab with the PD-1-AK105 complex structure. Nivolumab-Fab, pembrolizumab-Fab and AK015 are colored in slate, grey and yellow, respectively. PD-1 is shown as surface representation. (B) Binding surface of PD-1with AK105, pembrolizumab or nivolumab. The residues in contact with AK105 are colored in yellow, whereas residues in contact with nivolumab are colored in red, respectively, and the overlapping residues bounded by both AK105 and nivolumab are colored in orange. The residues in contact with pembrolizumab are colored in slate, and the overlapping residues bounded by both AK105 and pembrolizumab are colored in green.

**Supplementary Figure 4. The study flowchart for patients in the safety set**

**Supplementary Table 1. Test articles and dilution ratios in HPC residue test.**

| **Sample name** | **Subclass** | **Antibody concentration in preparation** | **Manufacturer** | **dilution ratio** |
| --- | --- | --- | --- | --- |
| Penpulimab | IgG1 | 10 mg/mL | Akeso | 2, 4, 8, 16, 32 |
| Pembrolizumab | IgG4 | 10 mg/mL | MSD | 2, 4, 8, 16, 32 |
| Nivolumab | IgG4 | 25 mg/mL | BMS | 2, 4, 8, 16, 32 |
| IgG4(A) | IgG4 | 10 mg/mL | commercially purchased | 15, 30，60, 120, 240 |
| IgG4(B) | IgG4 | 10 mg/mL |  | 2, 4, 8, 16, 32 |
| IgG4(C) | IgG4 | 40 mg/mL |  | 400, 800, 1600, 3200, 6400 |
| IgG4(D) | IgG4 | 40 mg/mL |  | 2, 4, 8, 16, 32 |

**Supplementary Table 2. Receptor Occupancy Analysis Sampling Schedule**

| Number of Doses (Dosing Cycle) | Days | Time relative to infusion of penpulimab |
| --- | --- | --- |
| Dose 1 (Cycle 1) | Day 1 | Before infusion (within 30 min before infusion) |
|  | Day 2 | 24 h (± 3 h) after the end of infusion |
|  | Day 8 | 168 h (± 1 day) after the end of infusion |
| Dose 2 (Cycle 1) | Day 15 | Before infusion (within 30 min before infusion) |
| Dose 3 (Cycle 2) | Day 29 | Before infusion (within 30 min before infusion) |
| Dose 5 (Cycle 3) | Day 57 | Before infusion (within 30 min before infusion) |
| Dose 9 (Cycle 5) | Day 113 | Before infusion (within 30 min before infusion) |
| Dose 13 (Cycle 7) | Day 169 | Before infusion (within 30 min before infusion) |
| Dose 17 (Cycle 9) | Day 225 | Before infusion (within 30 min before infusion) |
| Dose 21 (Cycle 11) | Day 281 | Before infusion (within 30 min before infusion) |
| Dose 25 (Cycle 13) | Day 337 | Before infusion (within 30 min before infusion) |
