# Supplementary figures and images for: Penpulimab, an Fc-Engineered IgG1 Anti-PD-1 Antibody, With Improved Efficacy and Low Incidence of Immune-Related Adverse Events
Source: Front Immunol. 2022 Jun 27;13:924542. doi: 10.3389/fimmu.2022.924542 (PMC9272907; doi:10.3389/fimmu.2022.924542)

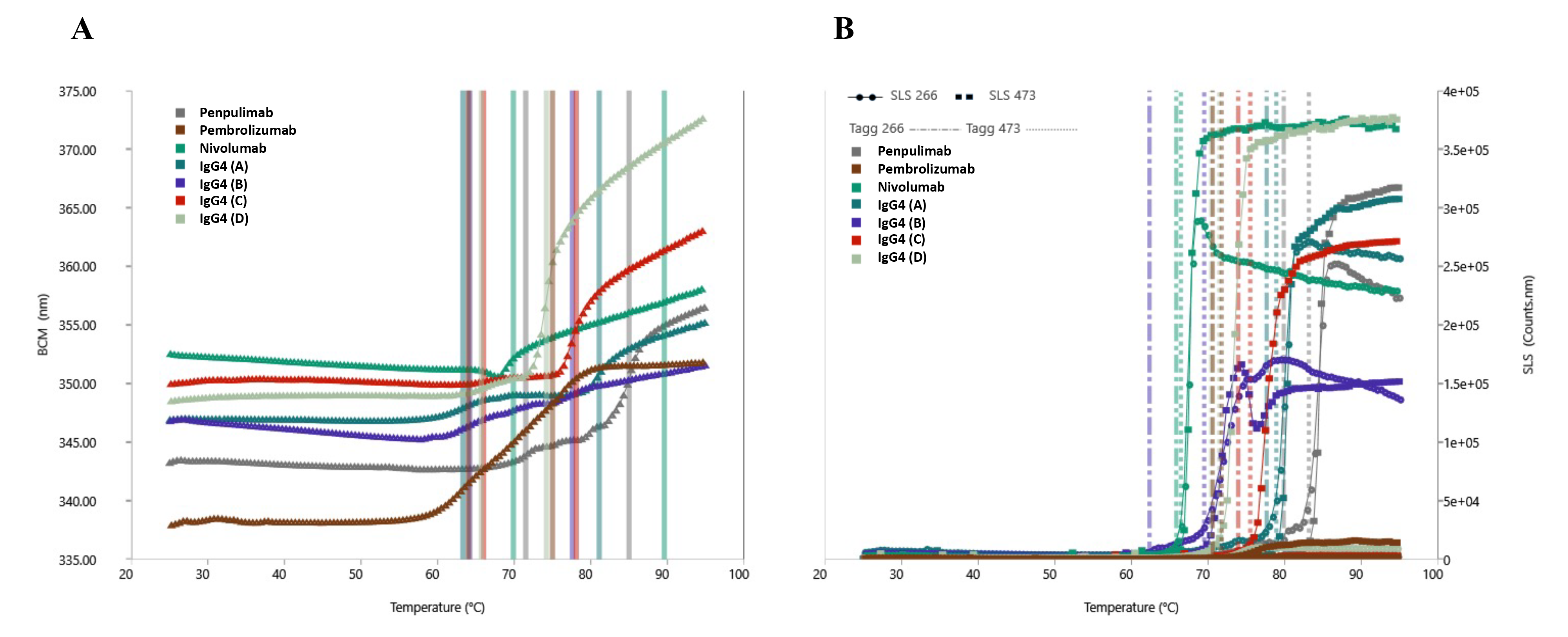

Supplement: Supplementary file 2 [file Image_1.tif]

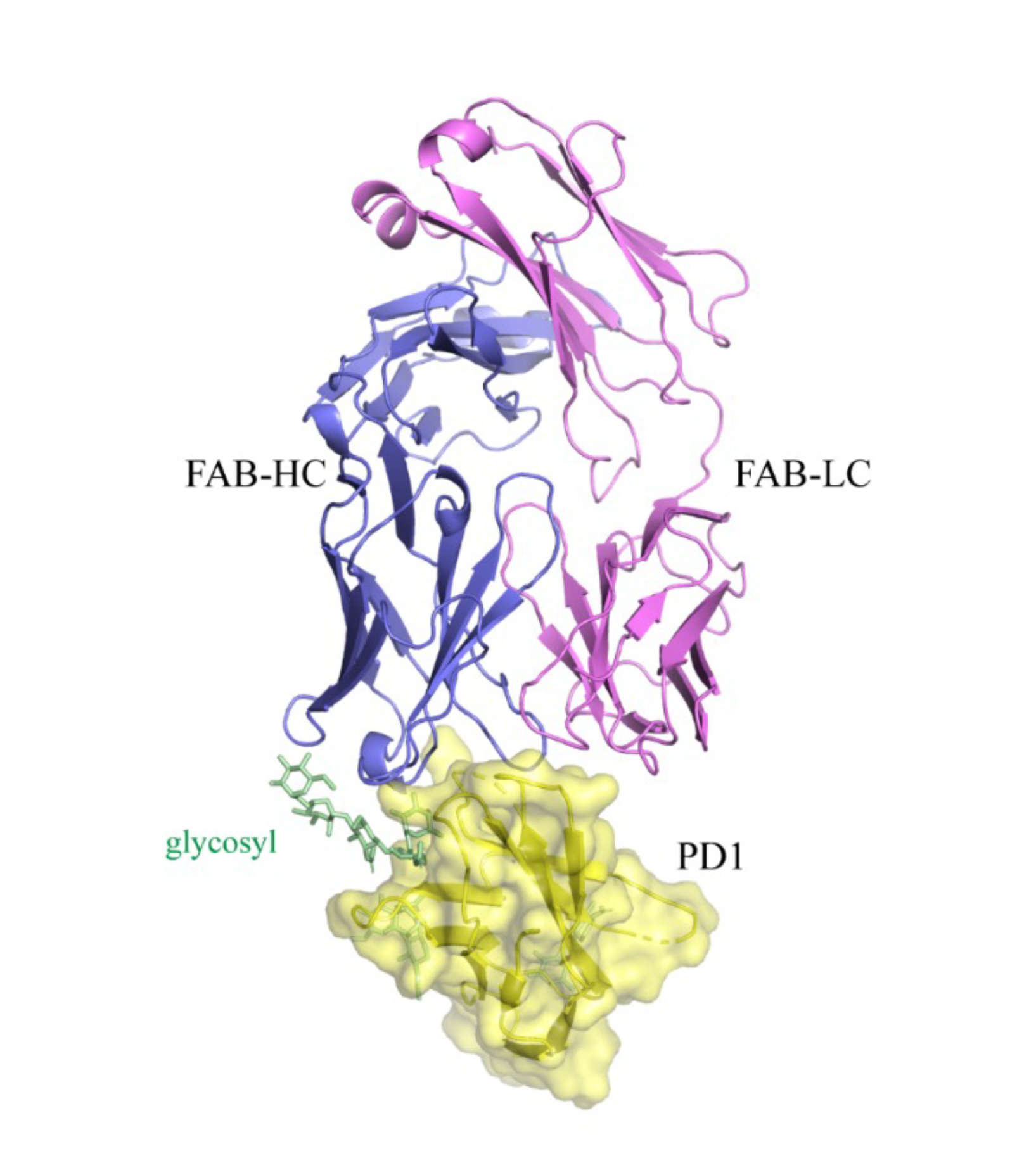

Supplement: Supplementary file 3 [file Image_2.tif]

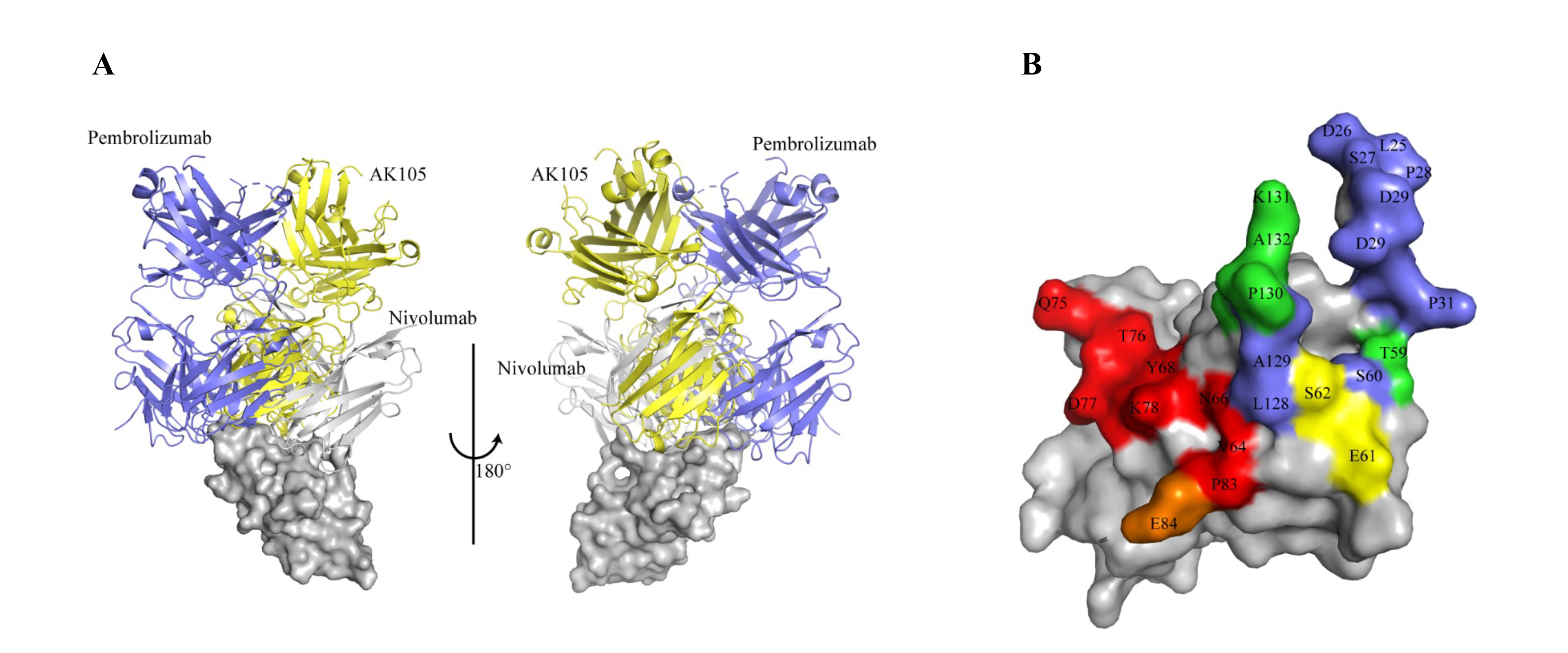

Supplement: Supplementary file 4 [file Image_3.tif]

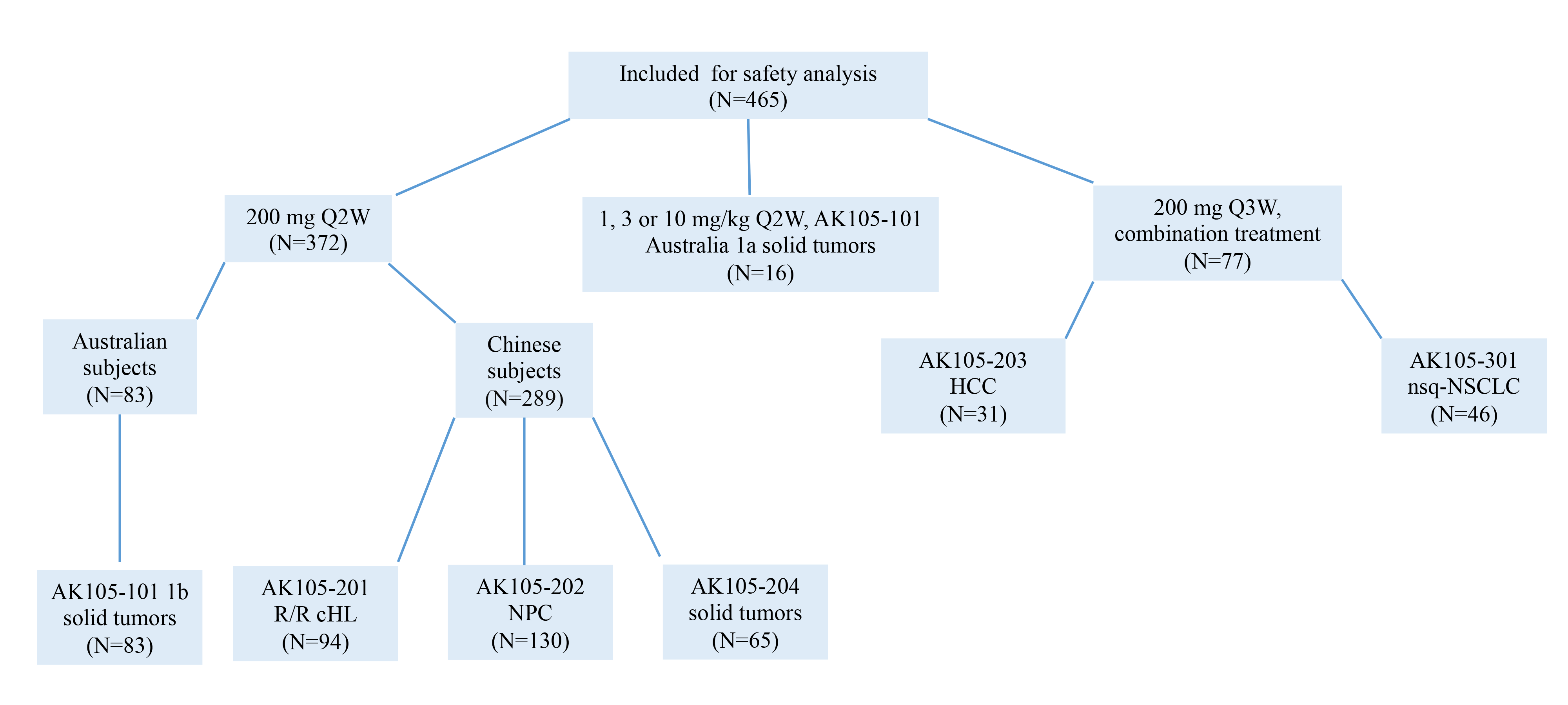

Supplement: Supplementary file 5 [file Image_4.tif]
